# Cytotoxic Necrotizing Factor-1 (CNF1) does not promote *E. coli* infection in a murine model of ascending pyelonephritis

**DOI:** 10.1186/s12866-017-1036-0

**Published:** 2017-05-25

**Authors:** Jason E. Michaud, Kwang Sik Kim, William Harty, Matthew Kasprenski, Ming-Hsien Wang

**Affiliations:** 1 James Buchanan Brady Urological Institute, Division of Pediatric Urology, The Johns Hopkins University School of Medicine, Baltimore, MD USA; 2 The Division of Pediatric Infectious Diseases, The Johns Hopkins University School of Medicine, Baltimore, MD USA

**Keywords:** Urinary tract infection, *E. coli*, pyelonephritis, Virulence factors

## Abstract

**Background:**

Urinary tract infections (UTI) are among the most common and costly infections in both hospitalized and ambulatory patients. Uropathogenic *E. coli* (UPEC) represent the majority of UTI isolates and are a diverse group of bacteria that utilize a variety of virulence factors to establish infection of the genitourinary tract. The virulence factor cytotoxic necrotizing factor-1 (CNF1) is frequently expressed in clinical UPEC isolates. To date, there have been conflicting reports on the role of CNF1 in the pathogenesis of *E. coli* urinary tract infections.

**Results:**

We examined the importance of CNF1 in a murine ascending kidney infection/ pyelonephritis model by performing comparative studies between a clinical UPEC isolate strain and a CNF1-deletion mutant. We found no alterations in bacterial burden with the loss of CNF1, whereas loss of the virulence factor fimH decreased bacterial burdens. In addition, we found no evidence that CNF1 contributed to the recruitment of inflammatory infiltrates in the kidney or bladder in vivo.

**Conclusions:**

While further examination of CNF-1 may reveal a role in UTI pathogenesis, our data casts doubt on the role of CNF-1 in the pathogenesis of UPEC UTI. As with other infections, different models and approaches are needed to elucidate the contribution of CNF1 to *E. coli* UTI.

**Electronic supplementary material:**

The online version of this article (doi:10.1186/s12866-017-1036-0) contains supplementary material, which is available to authorized users.

## Background

Urinary tract infections (UTI) are a major source of community and nosocomial infections and a major financial burden to healthcare systems [1]. Between 10 and 20% of United States women report at least one UTI and over 1 million hospitalized patients develop catheter-associated UTI annually [2, 3]. Accordingly, the United States spends an estimated 2.4 billion dollars annually on UTIs in adult women alone [2]. Likewise, febrile UTI are also a serious cause of bacterial infection in children and are associated with significant expenditures for hospital admissions due to febrile UTIs [4, 5].

The bladder is the most common site of UTI (cystitis) due to its proximity to the urethra and external intestinal flora. Most episodes of cystitis respond to antibiotic treatment, however, a subset of patients develop ascending acute pyelonephritis (APN), life-threatening bacterial urosepsis, or chronic, recurrent infections, often associated with antibiotic resistance. APN can lead to acute parenchymal damage and subsequent renal scarring in 15–60% of children and up to one third of adults [6, 7]. Ultimately, a subset of children with renal scarring will progress to hypertension, renal insufficiency, and less commonly end-stage renal disease [8].

Uropathogenic *E. coli* (UPEC) are the most common cause of UTI and are isolated in over 70% of cystitis or UTI-related bacteremia cultures [9, 10]. Epidemiological studies of clinical isolates have identified virulence factors that aid in colonization, host evasion, and survival of UPEC strains [11, 12]. UPEC exploit several virulence factors for adhesion, including P fimbriae and Type 1 fimbriae, which allow the attachment and ascent of bacteria within the genitourinary tract [12, 13]. Type 1 fimbriae bind mannosylated uroplakin receptors on urothelium, and this binding is essential to the ability of UPEC to colonize the bladder [13–15]. P fimbriae, or *pyelonephritis-associated* pili, are preferentially expressed in pyelonephritis isolates of UPEC and bind to glycosphingolipids present on renal epithelial cells [16, 17].

Other UPEC virulence factors include the secreted toxins α-hemolysin (HlyA*)* and Cytotoxic Necrotizing Factor 1 (CNF1). CNF1 and HlyA are commonly found in UPEC isolates and are co-expressed as closely linked genes [18, 19]. HlyA alters the cytoskeleton of urothelial cells resulting in shedding of bladder urothelium and disruption of urothelial barrier function [20, 21]. CNF1 is a 115-kDa secreted toxin with a well-characterized role in the pathogenesis of neonatal meningitis from *E. coli* K1 infection [22]. CNF1 is expressed in 31–44% of cystitis and 36–48% of pyelonephritis human clinical UPEC isolates [23–25]. Despite the common expression of CNF1 in UPEC isolates, a role of CNF1 in the pathogenesis of clinical UTI remains unclear. CNF1 has been shown to activate Rho GTPases, contribute to urothelial cell invasion, and have cytotoxic effects on urothelium [26, 27]. Studies utilizing CNF1-deficient mutants have provided mixed results. A clinical UPEC isolate has been shown to outcompete a CNF1-deleted UPEC strain in vivo in a mouse model of cystitis [28]. However, a similar CNF1-deletion mutant failed to reduce the cytotoxicity to urothelium in vitro [29]. Given the existing data supporting a role for CNF1 in the pathogenesis of UPEC cystitis, we sought to examine the potential role of CNF1 in *E. coli* pyelonephritis in vivo. To this end, we compared the ability of CNF1-deletion and wild-type UPEC to induce pyelonephritis in a mouse model of ascending infection.

## Methods

### Bacterial strains

The *E. coli* strain U8 was isolated previously from a female cystitis patient and has been characterized previously as an O18:K1:H7 serotype containing *cnf1*, *hlyA*, Type II capsule (*kpsMT*), OmpT (*ompT*), P- (*prf*), S- (*sfa*), and Type 1 (*fim*) fimbriae [30, 31]. *E. coli* strain RS218 (O18:K1:H7) was isolated previously from the cerebrospinal fluid of a neonate with meningitis [32]. The RS218 deletion mutants of *cnf1* and *fimH* have been described previously [22, 33]. *E. coli* stocks were stored at −70° in Luria-Bertani (LB) broth supplemented with 20% glycerol. The identity of bacterial strains were routinely checked by PCR and plating on LB agar with strain-appropriate antibiotic selection. Unless otherwise indicated, *E. coli* strains were routinely grown overnight at 37 °C in LB supplemented with the strain-appropriate antibiotics ampicillin (100 μg/ml), streptomycin (100 μg/ml), or chloramphenicol (20 μg/ml).

### Construction of isogenic *cnf1* deletion mutants

The *cnf1* deletion mutant of U8 (*Δcnf1) was* constructed using the one-step PCR method by replacing the *cnf1* gene with a chloramphenicol resistance cassette using phage λ Red recombinase as described previously [34]. Briefly, the U8 strain was transformed with pKD46 encoding arabinose-inducible λ Red recombinase. A chloramphenicol resistance cassette was amplified from pKD3 using primers with homologous extensions, 3′ homology to the chloramphenicol cassette and 5′ homology to the 5′ and 3′ regions of the *cnf1* gene: CNF1-KOF 5′-GAAAGGTGTCGCGTAATTTATCACCAGACCTTTGTTGATACATACTCAAAGTGTAGGCTGGAGCTGCTTC, CNF1-KOR 5′GGCTCATATCTTCTCCTGTCATGTGTGACTGCACTGTTTTGTGGCAAACCCATATGAATATCCTCCTTAG. The resulting PCR product was gel purified and transferred to competent and arabinose treated pKD46-containing U8 clones which expressed Red recombinase via electroporation. Transformants were grown on LB agar containing chloramphenicol (25 μg/ml) and 10 mM arabinose. The resultant deletion mutants of *cnf1* were confirmed with PCR using the primers CNF1-CKF 5′ AAATCGAAACGGCTCATCCG and CNF1-CKR 5′- CCTCTGGAAGAGTCTGTAAC. Lack of CNF1 protein expression was confirmed by Western blot using a monoclonal anti-CNF1 antibody as described previously [22]. For use in mixed-infection experiments, a spontaneous streptomycin-resistant mutant of the U8 parent strain was isolated using standard direct-selection techniques. Deletion mutants and parent strains demonstrated the same growth rates and characteristics in solid and liquid cultures.

### Murine model of pyelonephritis

All mice were housed in pathogen-free conditions, provided food and water ad libitum and all animal protocols were approved by an institutional animal care and use committee. The mouse strains C57BL/6, C3H/HeOuJ, and CBA/J were purchased from Jackson Labs, Bar Harbor, ME. Murine pyelonephritis and cystitis was established by transurethral inoculation of bacteria using a modification of previously described protocols [35, 36]. 6 to 8 week old mice were anesthetized with isoflurane, catheterized with 0.61 diameter polyethylene tubing (BD Biosciences*,* San Jose, CA), and transurethrally injected with 2 × 10^7^ CFU/ml of bacteria in 50 μl. Mice were allowed to recover for 3 h, and then underwent an identical second inoculation. As described previously, two transurethral instillations of *E. coli*, separated by three hours, produced a higher rate of pyelonephritis (80–90%), and more reproducible infections in C57BL/6 mice, when compared to a single instillation [36]. At designated times, mice were euthanized and the bladder and kidneys from each mouse were harvested and either homogenized in phosphate buffered saline (PBS) for bacterial enumeration or processed for flow cytometry. For bacterial enumeration, organs were homogenized and plated using serial dilution on LB agar to calculate the number of bacterial colony forming units (CFU) in each organ. For mixed-infections bacterial strains were plated on strain-appropriate antibiotics to obtain CFU for individual strains. The identity of bacterial strains isolated from animals was routinely checked by PCR and plating on LB agar with strain-appropriate antibiotic selection. Data from in-vivo experiments is displayed in Figures and available in a Additional file 1 online.

### Flow cytometry

Flow cytometric analysis of kidney and bladder infiltrates was performed on single cell suspensions isolated following digestion. Following infection, kidneys and bladders were homogenized in RPMI media (Thermo Fisher Scientific, Waltham, MA) containing 1 mg/ml Collagenase D and 100 μg/ml DNase I (Roche Diagnostics, Indianapolis, IN), supplemented with 20 mM HEPES and 10% fetal calf serum (FCS). Suspensions were and digested for 45 min at 37 °C, passed through 40 μm cell strainers, treated with BD Pharm Lyse™ (BD Biosciences, San Diego, CA), and cell pellets rinsed in PBS with 2% FCS. For cell staining 1 × 10^6^ cells were placed on ice in 12 × 75 mm tubes, blocked with Fc Block™ and stained with a cocktail of conjugated antibodies for 15 min, rinsed in PBS with 2% FCS, and stained with 3 μM DAPI (4′,6-diamidino-2-phenylindole, Thermo Fisher Scientific, Waltham, MA). Absolute cell numbers were determined using 123count eBeads (eBioscience, San Diego, CA) following the manufacturer’s instructions. Samples were analyzed with a BD LSR II flow cytometer and FACSDiva software (BD Biosciences, San Diego, CA). Antibodies used included: BV605 rat anti-mouse CD45, PerCP-Cy5.5 rat anti-mouse Ly-6C, FITC rat anti-mouse Ly-6G (BD Biosciences, San Diego, CA), and APC anti-mouse CD11c, PE/Cy7 anti-mouse CD11b, and PE anti-mouse F4/80 (Biolegend, San Diego, CA).

### Statistical analysis

All experiments were performed in triplicate to ensure reproducibility and representative data are shown. Data from groups of mice were compared using Students t-test, with *p* ≤ 0.05, considered significant.

## Results

### Effect of *cnf1* deletion on *E. coli* infection in the murine urinary tract

**Fig. 1:**
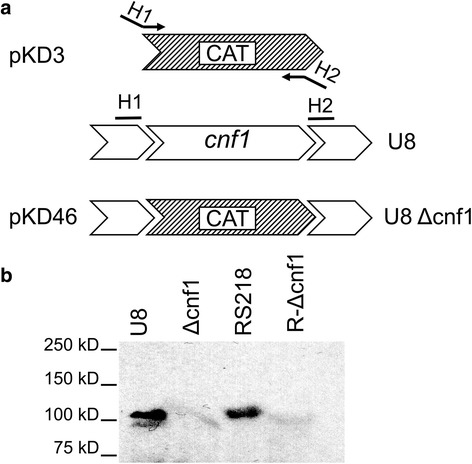
Construction of U8 *cnf1* deletion mutant. **a** One-stp PCR deletion of *cnf1* in U8 bacteria (Δcnf1). A chloramphenicol resistance cassette (CAT) was PCR-amplified from pKD3 with homologous extensions (H1 and H2) of the CNF1 locus and transferred to pKD46 Red recombinase-expressing U8 clones and selected for chloramphenicol resistance. **b** Western blot of U8, U8 *cnf1* deletion mutant (Δcnf1*),* RS218, and RS218 *cnf1* deletion mutants (R Δcnf1*)* with monoclonal CNF1 antibody

The *E. coli* virulence factor CNF1 has been previously implicated in the pathogenesis of UPEC bladder infection but its potential role in kidney infection remains unknown [28]. In order to examine the contribution of CNF1, we generated an isogenic *cnf1* deletion mutant (*Δcnf1)* of the clinical isolate UPEC strain U8 (O18:K1:H7). As shown in Fig. 1a, a one-step PCR method was utilized to delete the *cnf1* gene in the U8 strain [6]. The resultant cnf1-deletion mutant lacked CNF1 expression when compared to the parent U8 strain (Fig. 1b). Also shown in Fig. 1b is the *E. coli* strain RS218 (O18:K1:H7) and the RS218 deletion mutant of *cnf1* described previously [22, 33].

**Fig. 2:**
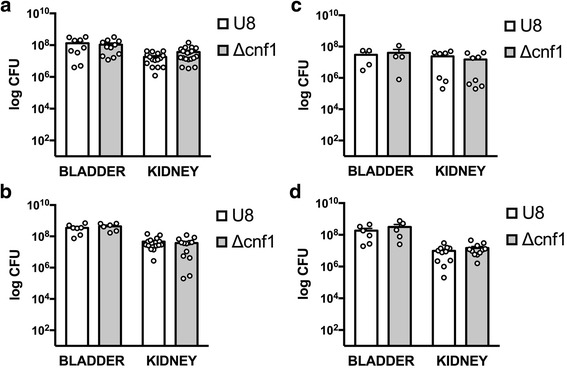
Bacterial counts (CFU/ml) in kidney and bladder during pyelonephritis. **a** 6 h, **b** 24 h, or **c** 10 days following infection with the parent strain U8, or *cnf1* deletion mutant (Δcnf1). **d** Mixed-infection model showing bacterial counts of U8 or Δcnf1 strains 24 h following infection with both strains in single animals

To examine the role of CNF1 in the pathogenesis of pyelonephritis, we used a murine model of ascending pyelonephritis following transurethral instillation of *E. coli* [35, 36]. As shown in Fig. 2, C57BL/6 mice developed reproducible kidney and bladder infections following transurethral instillation. When compared to the parent U8 strain, loss of *cnf1* did not alter bacterial counts in the kidney or bladder. In addition, the kinetics of infection with the *cnf1* deletion mutant were the same as those with the parent U8 strain, both early during infection at 6 h (Fig. 2a), 24 h (Fig. 2b), and late during infection at 10 days (Fig. 2c). To more directly compare the ability of U8 and *cnf1* deletion mutant strains to cause UTI in the same animal, we used a mixed-infection model. This model attempts to eliminate inter-animal variability by infecting the same animal with both strains, allowing competition between strains in a single animal. After introduction of U8 and *cnf1* deletion strains in equal amounts in the same animal, we found no difference in bacterial counts of either strain in either the bladder or kidney at 24 h (Fig. 2d).

**Fig. 3:**
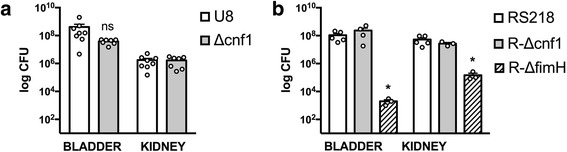
Bacterial counts (CFU/ml) in kidney and bladder during pyelonephritis, 24 h after infection. **a** CBA/J mice infected with the parent strain U8, or *cnf1* deletion mutant (Δcnf1). **b** C3H/HeOuJ mice infected with the parent RS218 or deletion mutants of *cnf1* (Δcnf1) or *fimH* (ΔfimH). (*p < 0.05) (ns, not significant)

There is known variability in the susceptibility among mouse strains to UTI [37]. Therefore, we performed similar experiments testing the effect of *cnf1* deletion on bacterial virulence in two additional mouse strains: C3H/HeOuJ and CBA/J. The CBA/J and C3H/HeOuJ strains have been used by others in UTI models [28, 35]. We found robust infections in the bladder and kidney of both C3H/HeOuJ and CBA/J mice (Fig. 3). Compared to C57BL/6 mice, CBA/J mice demonstrated a 1.4-log reduction in bacterial counts in the kidney at 24 h (not shown). However, we found no difference in bacterial counts between the parent U8 strain and the *cnf1* deletion mutant in infected CBA/J mice (Fig. 3a).

CNF1 has been shown previously to contribute to UTI in the C3H/HeOuJ strain, therefore, we next performed experiments in C3H/HeOuJ mice. Using the *E. coli* parent strain RS218 and an isogenic deletion mutants of *cnf1* (*R-Δcnf1),* deletion of *cnf1* did not alter the ability of UPEC to infect the bladder or kidney of C3H/HeOuJ mice (Fig. 3b) [28]. Given the apparent lack of contribution of CNF1 to infection in our model of acending pyelonephritis, we next examined the contribution of type I fimbriae in our model using a tip adhesin *fimH* (R-*ΔfimH)* mutant in the RS218 strain (Fig. 3b). FimH as is a well-documented UPEC virulence factor involved in bacterial adherence to urothelium [13, 14]. In contrast to data from *cnf1* deletion mutants, loss of *fimH* resulted in a 4.7 log-reduction and 2.5 log-reduction in bacterial counts within the bladder and kidney, respectively (Fig. 3b, *p*=0.04 and *p* = 0.03).

### Role of CNF1 in leukocyte recruitment during pyelonephritis

**Fig. 4:**
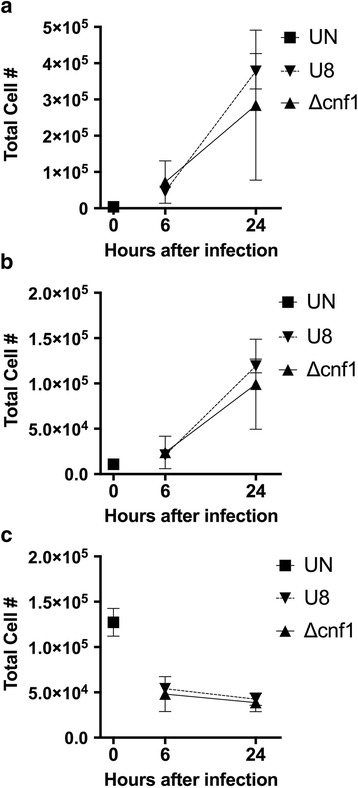
Flow cytometry analysis of kidney infiltrates during pyelonephritis infection with the parent U8 strain, or *cnf1* deletion mutant (Δcnf1). (UN, uninfected control) The number of (**a**) neutrophils (CD11b^+^, Ly6G^+^, Ly6C^lo^), (**b**) monocytes (CD11b^+^, Ly6G^−^, Ly6C^hi^), and (**c**) renal dendritic cells (CD11b^+^, Ly6G^−^, Ly6C^lo^, CD11C^+^) are shown

**Fig. 5:**
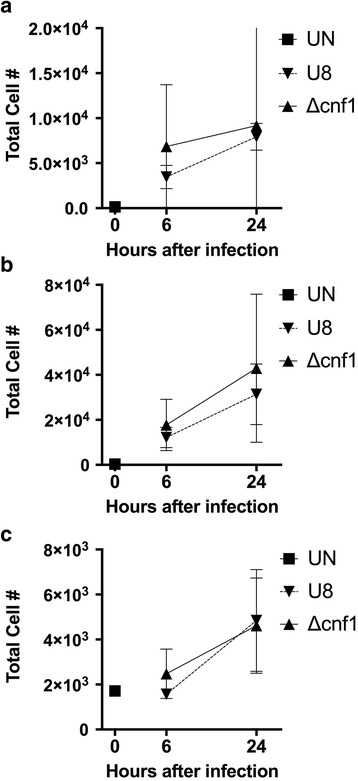
Flow cytometry analysis of bladder infiltrates during pyelonephritis infection with the parent U8 strain, or *cnf1* deletion mutant (Δcnf1). (UN, uninfected control) The number of (**a**) neutrophils (CD11b^+^, Ly6G^+^, Ly6C^lo^), (**b**) monocytes (CD11b^+^, Ly6G^−^, Ly6C^hi^), and (**c**) myeloid dendritic cells (CD11b^+^, Ly6G^−^, Ly6C^lo^, CD11C^+^) are shown

CNF1 has been shown to upregulate genes related to innate immunity in the mouse bladder during cystitis and to modulate neutrophil function in vitro [38, 39]. Based on these findings, we explored the potential role of CNF1 to modulate leukocyte recruitment during pyelonephritis. We used flow cytometry to characterize the leukocyte composition of the kidney and bladder in mice with pyelonephritis. Infection with the parent UPEC strain U8 in our model of ascending pyelonephritis induced rapid recruitment of neutrophils and Ly6C^hi^inflammatory monocytes to the kidney (Fig. 4a, b) and bladder (Fig. 5a, b) within 6 h after infection. Deletion of *cnf1* did not alter the robust neutrophil and monocyte infiltrate in mice infected with the *cnf1* deletion mutant UPEC in either the kidney (Fig. 4a, b) or bladder (Fig. 5a, b).

We also examined kidney and bladder dendritic cells (DC) during pyelonephritis. Renal DC are resident cells important for innate immunity and bacterial clearance during pyelonephritis [36, 40]. In addition, CNF1 has recently been shown to alter *E. coli* uptake by macrophages [41]. In response to renal injury, renal DC migrate to draining LNs where they present antigens [42]. As such, we observed a rapid decrease in renal DC during pyelonephritis (Fig. 4c). We also found equivalent numbers of renal DC in mice infected with either U8 *E. coli* or the *cnf1* deletion mutant (*Δcnf1)* (Fig. 4c). Unlike renal DC, the majority of bladder DC during UTI are recruited to the bladder and are less important for bacterial clearance [43]. We observed no statistically significant difference in the numbers of DC recruited to bladders of mice infected with U8 *E. coli* or the *cnf1* deletion mutant (*Δcnf1)* (Fig. 5).

## Discussion

In this study, we examined the role of CNF1 in the pathogenesis of *E. coli* UTI in vivo using a mouse model of pyelonephritis by transurethral inoculation. Using the UPEC strain U8 and an isogenic *cnf1* deletion mutant, we demonstrated that *E. coli* lacking CNF1 were equally efficient at infecting the bladder and kidney after transurethral inoculation. We found no evidence that deletion of *cnf1* altered the severity of UPEC during UTI. Our results are in agreement with prior studies by Johnson et al., who found that deletion of *cnf1* did not alter UPEC virulence during UTI [44]. In contrast, Rippere-Lampe and colleagues have previously described attenuation of UPEC virulence after *cnf1* deletion [28]. In addition, recent data suggests that antibodies directed against CNF1 can reduce bacterial burden during experimental models of cystitis [45].

There are several notable differences between our study and those of other groups. *First*, differences in animal models of UTI will directly affect the bacterial-host interaction. We specifically designed our model to cause robust pyelonephritis in the majority of animals. To accomplish this our group, and others, used two transurethral inoculations of UPEC, compared to a single inoculation with standard UTI models [35–37]. We use primarily C57BL/6 mice, but also performed experiments in the C3H/HeOuJ strain used by Rippere-Lampe and colleagues. *Second*, we used different clinical UPEC isolates, which may vary in their ability to infect the urinary tract. In an attempt to mitigate strain-specific findings we obtained similar results with *cnf1* deletion mutants from two *E. coli* strains, RS218 and U8. It is possible that there is variation between UPEC strains containing CNF1, and that CNF1 plays a role in infection in other strains. It is also possible that certain host mouse strains are more susceptible to the effects of CNF1 expression in UPEC. In our model, however, we found no evidence to support the hypothesis that CNF1 plays a significant role in the infection of the urinary tract in vivo*.*

The cytotoxic effects of CNF-1 have been well documented. CNF-1 induces stress fiber formation in endothelial cells to promote bacterial invasion and has been shown to be directly cytotoxic to urothelium [22, 26, 27]. In addition, CNF1 has been shown to upregulate genes related to innate immunity in the mouse bladder during cystitis, and to modulate neutrophil function in vitro [38, 39]. Despite these findings, we did not find a role for CNF-1 in altering immune cell infiltration or DC migration during pyelonephritis. However, this does not exclude a role for CNF-1 in regulating inflammation during cystitis or specific DC subsets during immune responses.

A role for CNF-1 in the pathogenesis of UTI is often inferred from the prevalence of CNF-1 expression in clinical isolates of UPEC. Yet, there is strong evidence that the virulence of *cnf1* expressing strains is derived from the co-expression of *hlyA.* Prior CNF-1 studies have highlighted the close genetic linkage between *hly* and *cnf1* [18, 19]. *Cnf1* is expressed in 31–44% of cystitis and 36–48% of pyelonephritis clinical isolates [23–25], whereas approximately one-half of *E. coli* isolated from cystitis (40%) or pyelonephritis (49%) isloates produce hemolysin [24]. Of hemolytic isolates from extraintestinal infections (primarily UTI strains), a large fraction (65 to 83%) encode the *cnf1*, but *cnf1* is rarely found in nonhemolytic UPEC isolates [1, 5, 8, 22]. Furthermore, animal studies, including ours, have failed to provide *hly*-independent effects of *cnf1* using deletion mutants.

## Conclusions

While further examination of CNF-1 may reveal a role in UTI pathogenesis, our data casts doubt on the role of CNF-1 in the pathogenesis of UPEC UTI. Future research on *E. coli* virulence factors is clearly needed, as urologic infections are an important and evolving clinical problem. In the era of rising antibiotic resistance, understanding bacterial virulence mechanisms may reveal further therapeutic targets and lead to novel treatments.

## Additional file

Additional file 1: Bacterial and flow cytometry counts from in-vivo pyelonephritis model. (XLSX 54 kb)

## Abbreviations

CNF1: Cytotoxic necrotizing factor-1
DC: Dendritic cells
UPEC: Uropathogenic *E. coli*
UTI: Urinary tract infections
